# Supplementary material for: CD163-Mediated Small-Vessel Injury in Alzheimer’s Disease: An Exploration from Neuroimaging to Transcriptomics
Source: Int J Mol Sci. 2024 Feb 14;25(4):2293. doi: 10.3390/ijms25042293 (PMC10888773; doi:10.3390/ijms25042293)
Supplement: Supplementary file 1 [file ijms-25-02293-s001.zip › ijms-2842076-supplementary.pdf]

Table S1: The DTI indices results of comparison among CN, MCI, Dementia

FA_ROI	FA_p_adj_value	RD_ROI	RD_p_adj_value	AD_ROI	AD_p_adj_value	MD_ROI	MD_p_adj_value
FA_TAP_L	0.014725	RD_CGH_L	1.37E-05	AD_CGH_L	4.22E-05	MD_CGH_L	1.27E-05
FA_TAP_R	0.014725	RD_SS_L	3.11E-05	AD_SCC_L	6.17E-05	MD_SS_L	2.57E-05
FA_FX_L	0.014725	RD_CGC_L	0.00011058	AD_SS_L	6.17E-05	MD_CGC_L	1.09E-04
FA_FX_ST_L	0.014725	RD_SCC_L	0.00045315	AD_SS_R	6.17E-05	MD_SCC_L	1.42E-04
FA_PTR_L	0.014725	RD_UNC_L	0.00054815	AD_SUMSCC	6.34E-05	MD_SUMSCC	2.16E-04
FA_SCC_L	0.014725	RD_SUMSCC	0.00054815	AD_PCR_L	6.34E-05	MD_SS_R	2.16E-04
FA_SUMSCC	0.019787143	RD_PTR_L	0.000830571	AD_UNC_L	7.50E-05	MD_UNC_L	2.16E-04
FA_SCC_R	0.032205	RD_SS_R	0.0008322	AD_SUMCC	1.51E-04	MD_PCR_L	2.69E-04
FA_SUMFX	0.03667	RD_FX_ST_L	0.0008322	AD_SCC_R	5.42E-04	MD_SUMCC	4.68E-04
FA_SUMCC	0.05871	RD_TAP_L	0.0008322	AD_FX_ST_L	0.0005415	MD_PTR_L	4.79E-04
FA_SUMGCC	0.078046154	RD_SCC_R	0.000933923	AD_PTR_L	0.0005415	MD_SCC_R	4.79E-04
FA_CGC_L	0.078046154	RD_SUMCC	0.000933923	AD_SCR_L	0.0005415	MD_GCC_L	0.0005035
FA_GCC_R	0.078046154	RD_FX_L	0.000933923	AD_GCC_L	0.0005415	MD_FX_ST_L	0.000513
FA_ACR_L	0.08398	RD_GCC_L	0.001127786	AD_TAP_L	0.0005415	MD_TAP_L	0.000513
FA_GCC_L	0.08398	RD_IFO_R	0.0012046	AD_BCC_L	0.0005814	MD_IFO_R	0.000580688
FA_SS_L	0.086505882	RD_IFO_L	0.00121125	AD_CGC_L	0.000630563	MD_IFO_L	0.000580688
FA_SFO_R	0.086505882	RD_SUMGCC	0.001542353	AD_FX_ST_R	0.000690706	MD_SUMGCC	0.000924667
FA_FX_R	0.108616667	RD_TAP_R	0.001908	AD_IFO_R	0.001102	MD_FX_L	0.000924667
FA_CGC_R	0.1365	RD_PCR_L	0.001908	AD_SUMBCC	0.002358	MD_SCR_L	0.000972
FA_CGH_L	0.159885	RD_ACR_L	0.002890714	AD_SUMGCC	0.0025365	MD_BCC_L	0.0020007
FA_PTR_R	0.168285714	RD_SUMFX	0.002890714	AD_IFO_L	0.003002	MD_SUMFX	0.002292391
FA_CGH_R	0.17385	RD_GCC_R	0.0033345	AD_SUMFX	0.003059864	MD_TAP_R	2.29E-03
FA_FX_ST_R	0.178930435	RD_UNC_R	0.003955304	AD_PCR_R	0.003087913	MD_FX_ST_R	0.002292391
FA_ACR_R	0.1812125	RD_SCR_L	0.004218	AD_RLIC_R	0.003465125	MD_GCC_R	0.0031825
FA_UNC_R	0.220248	RD_FX_ST_R	0.0042294	AD_FX_L	0.00365484	MD_UNC_R	0.0035112
FA_BCC_R	0.223615385	RD_BCC_L	0.005888538	AD_UNC_R	0.003972462	MD_ACR_L	0.004621385

FA_CP_L	0.240214286	RD_ACR_R	0.006120111	AD_RLIC_L	0.005095393	MD_SUMBCC	0.004688778
FA_CP_R	0.240214286	RD_CGC_R	0.008301643	AD_TAP_R	0.005095393	MD_PCR_R	0.006017571
FA_IFO_R	0.241758621	RD_SUMBCC	0.009994655	AD_SCR_R	0.005912276	MD_ACR_R	0.006509793
FA_SUMBCC	0.2831	RD_FX_R	0.0128421	AD_FX_R	0.0063251	MD_CGC_R	0.0072276
FA_IFO_L	0.36159375	RD_SCR_R	0.014409968	AD_BCC_R	0.011810032	MD_SCR_R	0.007373226
FA_ICP_R	0.36159375	RD_CGH_R	0.015521813	AD_CGH_R	0.015021281	MD_FX_R	0.008462719
FA_BCC_L	0.378272727	RD_BCC_R	0.015567909	AD_CGC_R	0.016243324	MD_BCC_R	0.011315364
FA_ICP_L	0.404029412	RD_PCR_R	0.022546853	AD_GCC_R	0.016243324	MD_CGH_R	0.012224824
FA_ALIC_R	0.434828571	RD_CP_L	0.026742771	AD_ACR_R	0.018943	MD_RLIC_R	0.015489343
FA_SS_R	0.474486486	RD_PTR_R	0.03854625	AD_SLF_L	0.018943	MD_RLIC_L	0.02901775
FA_SCP_L	0.474486486	RD_SFO_L	0.042497351	AD_ACR_L	0.028960622	MD_SLF_L	0.032197297
FA_SFO_L	0.5175	RD_SFO_R	0.0649395	AD_EC_L	0.0333675	MD_CP_L	0.038780462
FA_RLIC_L	0.532	RD_RLIC_R	0.073487615	AD_PLIC_L	0.035687846	MD_SFO_L	0.038780462
FA_SCP_R	0.56715	RD_ALIC_L	0.07954635	AD_SFO_L	0.0373065	MD_PTR_R	0.04015365
FA_CST_R	0.57	RD_SLF_L	0.080343585	AD_SLF_R	0.062964146	MD_PLIC_R	0.051263854
FA_SCR_L	0.599857143	RD_PLIC_R	0.082890214	AD_PTR_R	0.071465786	MD_SFO_R	0.070851
FA_PCR_L	0.615069767	RD_CP_R	0.105032442	AD_PLIC_R	0.089003512	MD_ALIC_L	0.07953886
FA_PLIC_R	0.746066667	RD_RLIC_L	0.166390435	AD_SFO_R	0.099952091	MD_EC_L	0.086325205
FA_ALIC_L	0.746066667	RD_ALIC_R	0.166390435	AD_CP_L	0.107149867	MD_PLIC_L	0.087733133
FA_ML_R	0.752152174	RD_EC_L	0.166390435	AD_ML_L	0.128604391	MD_SLF_R	0.128447022
FA_UNC_L	0.773744681	RD_SLF_R	0.255165957	AD_EC_R	0.138028532	MD_CP_R	0.12885517
FA_ML_L	0.811959184	RD_EC_R	0.281210688	AD_ALIC_L	0.13847675	MD_ALIC_R	0.174547063
FA_CST_L	0.811959184	RD_PLIC_L	0.342429176	AD_ALIC_R	0.185530347	MD_EC_R	0.200460857
FA_SLF_L	0.86526	RD_ICP_L	0.342429176	AD_CP_R	0.25668696	MD_ML_L	0.26073966
FA_SLF_R	0.917277778	RD_ML_R	0.342429176	AD_ML_R	0.344039706	MD_ML_R	0.307203176
FA_SCR_R	0.917277778	RD_ML_L	0.452940635	AD_ICP_L	0.398028808	MD_ICP_L	0.351784269
FA_EC_R	0.917277778	RD_ICP_R	0.531486283	AD_CST_R	0.497684208	MD_CST_L	0.543622981
FA_EC_L	0.917277778	RD_CST_L	0.551758944	AD_CST_L	0.503384944	MD_ICP_R	0.549085145

FA_PCR_R	0.927545455	RD_CST_R	0.587256491	AD_ICP_R	0.578548964	MD_CST_R	0.549085145
FA_RLIC_R	0.964928571	RD_SCP_R	0.678130018	AD_SCP_L	0.654596143	MD_SCP_L	0.701724964
FA_PLIC_L	0.979	RD_SCP_L	0.67978	AD_SCP_R	0.856957	MD_SCP_R	0.752549

one-way ANOVA was conducted to evaluate the comparison of DTI indices among three groups, and False Discovery Rate (FDR) correction was applied during the statistical analysis for the large number of multiple comparisons. Additionally, covariate adjustment, including age, gender and education level, was performed on the results to control for potential confounding factors, $p < 0.05$ was considered to be statistically significant. Abbreviations: refer to ADNI_DTIROI_V1_DICT (Download Study Data (usc.edu)).

Table S2: Demographic characteristics, neuropsychological tests and neuroimaging for four groups

	CN (n=149)		MCI (n=304)		*p*
	WMH- (n=75)	WMH+ (n=74)	WMH- (n=152)	WMH+ (n=152)	
Demographic characteristics					
Male, n (%)	36 (48.0%)	32 (43.2%)	79 (52.0%)	84 (55.3%)	0.360
Age, mean (SD)	71.94 (5.46)^{bcd}	74.97 (6.16)^{acd}	67.87 (6.43)^{abd}	75.05 (6.58)^{abc}	<0.001
Education, mean (SD)	16.64 (2.48)	16.73 (2.60)	16.24 (2.55)	15.95 (2.73)	0.107

APOEε4 carriers, n (%)	54/20/1 (72.0%/26.7%/1.3%)	51/18/5 (68.9%/24.3%/6.8%)	87/53/12 (57.2%/34.9%/7.9%)	88/53/11 (57.9%/34.9%/7.2%)	0.157
Neuropsychological tests					
MMSE	29.25 (0.95)[cd]	28.81 (1.39)[cd]	27.63 (2.37)[ab]	27.17 (2.84)[ab]	<0.001
FAQ	0.18 (0.67)[cd]	0.14 (0.53)[cd]	2.99 (4.58)[ab]	4.72 (6.34)[ab]	<0.001
MCAO	25.95 (2.31)[cd]	25.5 (2.00)[cd]	23.44 (3.77)[abd]	22.02 (4.01)[abc]	<0.001
CDRSB	0.03 (0.14)[cd]	0.04 (0.14)[cd]	1.30 (0.80)[ab]	1.45 (1.92)[ab]	<0.001
Neuroimaging					
ln_WMH_TCV	-2.21 (0.83)[bd]	-0.47 (0.68)[ac]	-2.20 (0.74)[bd]	-0.26 (0.68)[ac]	<0.001
TCB_TCV	78.14 (2.42)[d]	77.28 (2.56)[c]	78.42 (3.03)[d]	76.70 (2.77)[abc]	<0.001
T_hippo_TCV	0.57 (0.05)[d]	0.56 (0.06)[d]	0.55 (0.07)[d]	0.52 (0.07)[abc]	<0.001
FDG	1.30 (0.04)[d]	1.30 (0.06)[d]	1.28 (0.07)	1.27 (0.07)[ab]	0.001
AV45	1.10 (0.18)[d]	1.11 (0.18)[d]	1.16 (0.21)[d]	1.24 (0.23)[abc]	<0.001
TAU	247.43 (96.21)	238.85 (88.98)	256.8 (122.84)	279.68 (118.46)	0.073
PTAU	22.39 (9.50)	21.92 (9.09)[d]	24.32 (13.40)	26.65 (13.30)[b]	0.036

The $\chi 2$-test for gender and APOEε4, one-way ANOVA were performed to assess group comparison for another indices, Bonferroni correction for homoscedasticity and Tamhane's T2 test for heteroscedasticity, $p < 0.05$ was considered to be statistically significant. *a:* Significantly different from NC_WMH-; *b:* Significantly different from NC_WMH+; *c:* Significantly different from MCI _WMH-. *d:* Significantly different from MCI_WMH-. Abbreviations: CN, cognitively normal; MCI, mild cognitive impairment; SD, standard deviation; MMSE, Mini Mental State Examination; FAQ, Functional Activities Questionnaire; MoCA, Montreal Cognitive; CDRSB, Clinical Dementia Rating Scale Sum of Boxes; ln_WMH_TCV, Natural Logarithm of standardized white matter hyperintensities (WMH) to Total Cerebrum Cranial Volume (TCV); TCB_TCV, Standardized Total Cerebrum Brain Volume (TCB) to Total Cerebrum Cranial Volume (TCV); T_hippo_TCV, Standardized Segmented Total Hippocampi Volume (T_hippo) to Total Cerebrum Cranial Volume (TCV).

Table S3： The correlation of neuroimaging and the feature-selection genes

p value	CD163	ZNF23	FOLR2	ALDH3B1	MIR22HG	DTX2	ALDH2
FA_PTR_L	0.008746838	0.136646768	0.308086955	0.195202472	0.768759481	0.362138802	0.010205827
FA_FX_ST_L	0.00622964	0.124021567	0.040115339	0.067091162	0.550629104	0.625941228	0.063401248
FA_FX_L	0.01082901	0.353898847	0.04785647	0.120357859	0.897050392	0.306090076	0.061171114
FA_SCC_L	0.364385704	0.772129905	0.092650411	0.336926341	0.824122132	0.872114454	0.123016327
FA_SCC_R	0.396444317	0.7964025	0.085094866	0.414612907	0.796334338	0.760200225	0.082791347

FA_TAP_L	0.046810587	0.36837448	0.048438185	0.132814845	0.94733702	0.856826881	0.141602823
FA_TAP_R	0.332632355	0.372078691	0.047366183	0.063640615	0.786169124	0.244762009	0.325146406
FA_SUMSCC	0.372560753	0.788476693	0.085340738	0.36633678	0.812962131	0.809103169	0.098279648
FA_SUMFX	0.045136773	0.093238995	0.010237025	0.006264485	0.742613116	0.048008331	0.108994372

r value	CD163	ZNF23	FOLR2	ALDH3B1	MIR22HG	DTX2	ALDH2
FA_PTR_L	-0.344208924	0.199574944	-0.137404702	-0.174115672	-0.03980746	-0.122969634	-0.337666197
FA_FX_ST_L	-0.35814871	0.206099683	-0.272732793	-0.244253691	0.080709386	-0.065954828	-0.247524127
FA_FX_L	-0.335116329	0.12507915	-0.263266538	-0.208086925	-0.017524849	-0.137967661	-0.249575586
FA_SCC_L	-0.122399498	0.039211089	-0.224850014	-0.129521718	0.030097378	-0.021801917	-0.206640448
FA_SCC_R	-0.114485252	0.034937283	-0.230092414	-0.110166958	0.034949236	-0.041325368	-0.231763302
FA_TAP_L	-0.264468545	0.121392611	-0.262607252	-0.201505452	0.008946743	-0.024434306	-0.197137431
FA_TAP_R	-0.130667661	0.120463449	-0.26382731	-0.247307402	-0.036734762	-0.156581605	-0.132687895
FA_SUMSCC	-0.12034294	0.036328901	-0.229916177	-0.121906157	0.032041104	-0.032714693	-0.221157341
FA_SUMFX	-0.266438762	0.224455858	-0.337535386	-0.357924418	0.04446045	-0.263093798	-0.214559698

The correlation matrices between FA value and feature-selection genes. The first matrix contains p values, indicating the significance of the correlations. The second matrix presents r values, representing the strength and direction of the linear relationship between the variables. Abbreviations: refer to ADNI_DTIROI_V1_DICT (Download Study Data (usc.edu)).

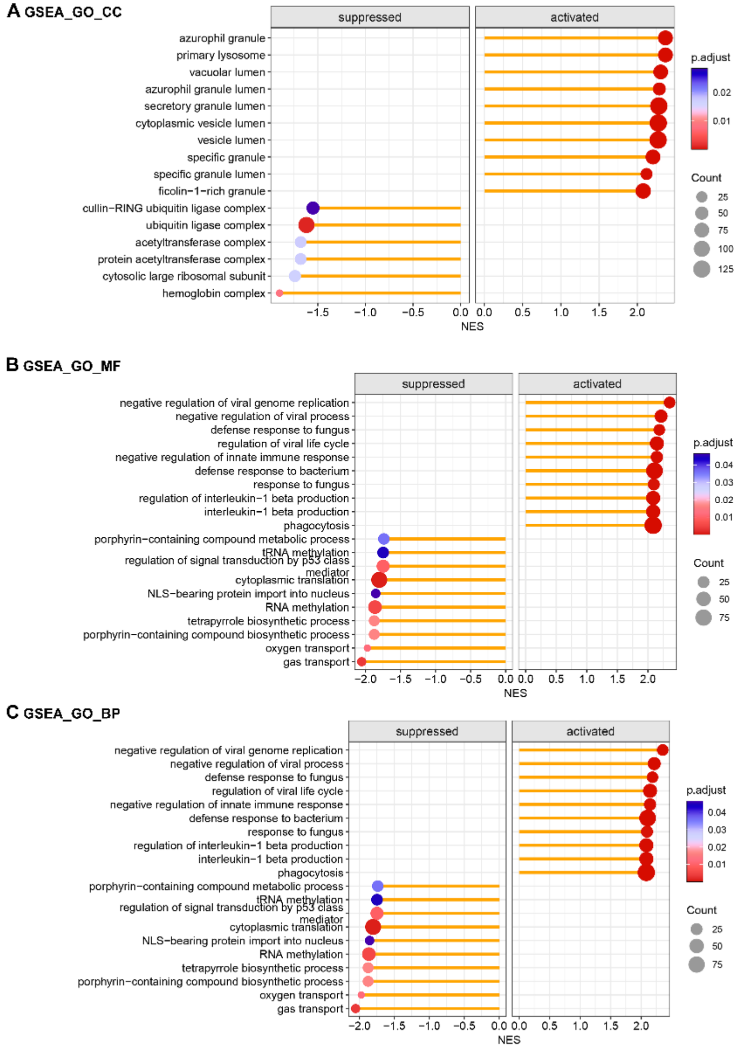

Figure S1: Gene Set Enrichment Analysis of Gene Ontology: including (**A**) biological process (BP), （**B**） cellular component (CC), and (**C**) molecular function (MF).

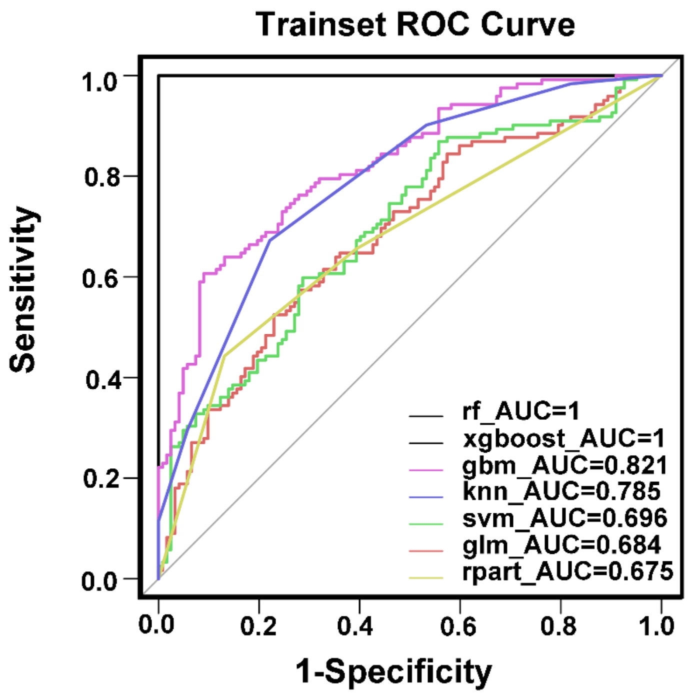

Figure S2: The predictive effectiveness of seven machine learning methods in the training set.

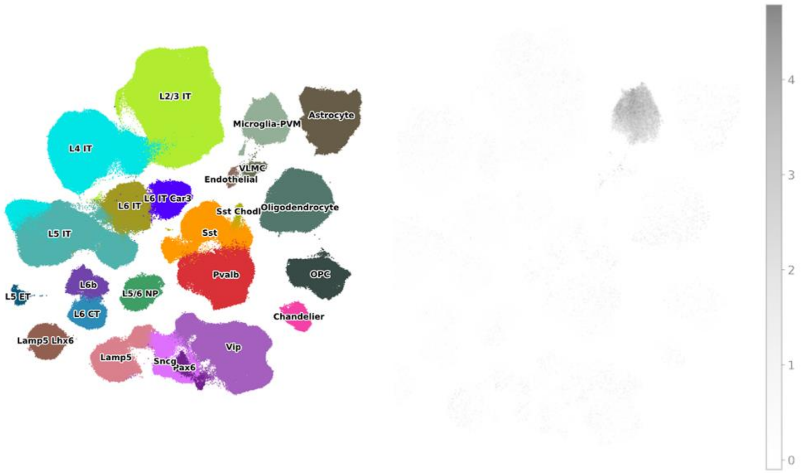

Figure S3: The expression of CD163 in different cells from brain in a public database (Seattle Alzheimer's Disease Brain Cell Atlas (SEA-AD)).

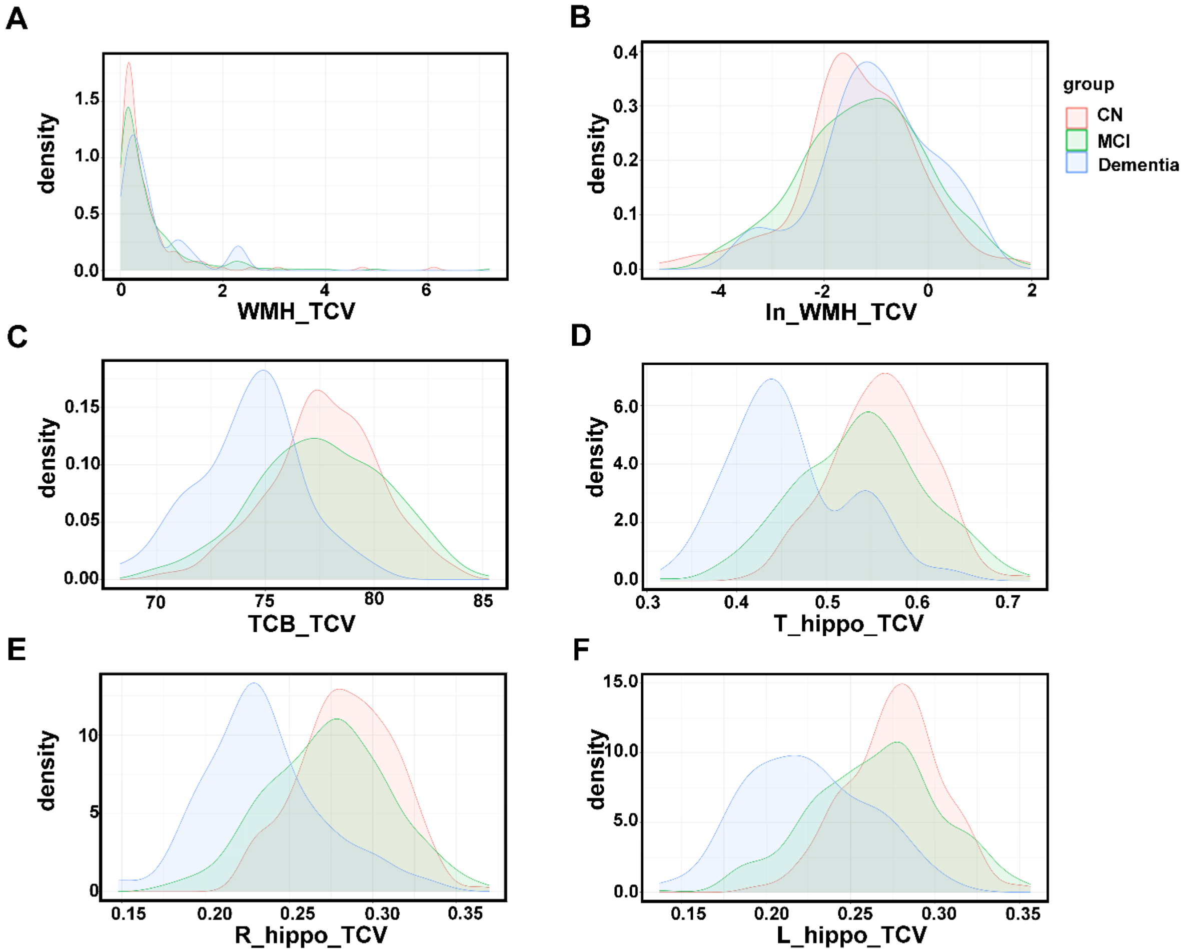

Figure S4: the distribution of data after transformation in preprocessing:
(**A, C-F**) the distribution of Total Cerebrum Brain Volume (TCB) and white matter hyperintensities (WMH) standardized by Total Cerebrum Cranial Volume (TCV) and subsequently multiplied by 100. (**B**) the distribution of WMH_TCV after natural logarithm transformed.
